# Integrating Multi-Source Directed Gene Networks and Multi-Omics Data to Identify Cancer Driver Genes Based on Graph Neural Networks

**DOI:** 10.3390/ijms262412132

**Published:** 2025-12-17

**Authors:** Yuetong Jiang, Yunjiong Liu, Ruoyao Qi, Shaowei Li, Tianying Zhang

**Affiliations:** 1 Institute of Artificial Intelligence, School of Informatics, Xiamen University, Xiamen 361005, China; 2 State Key Laboratory of Vaccines for Infectious Diseases, Xiang An Biomedicine Laboratory, School of Public Health, Xiamen University, Xiamen 361005, China; 3 National Institute of Diagnostics and Vaccine Development in Infectious Diseases, Xiamen University, Xiamen 361005, China

**Keywords:** cancer driver genes, graph neural network, directed graphs, multi-omics data

## Abstract

Precisely identifying cancer drivers helps us to understand the molecular mechanisms of cancer, offering critical targets for early diagnosis. Despite the increasing application of graph neural networks in predicting cancer driver genes, existing approaches do not fully leverage the information from gene networks, and are unable to effectively extract node features from directed graphs. To this end, we propose MDIGNN, a novel deep learning model designed to identify cancer driver genes by integrating directed gene networks with multi-omics data. First, we construct a directed graph through the integration of existing gene networks from diverse databases and multi-omics data. Then, to encode the edge directionality, we develop a graph neural network based on the magnetic Laplacian, which relies on a complex Hermitian matrix for representing the directed graph structure. Next, we apply the channel attention and spatial attention mechanisms to improve the model’s feature representation ability. Finally, MDIGNN uses a fully connected layer to compute the cancer driver probability for each gene. In a comparative evaluation, MDIGNN outperforms existing state-of-the-art methods in the field, and it is capable of detecting potential cancer driver genes.

## 1. Introduction

Cancer is a complex disease characterized by uncontrolled cellular growth and proliferation driven by genetic alterations. In this process, cancer driver genes play a critical role, which provide a growth advantage to tumor cells through specific mutations or abnormal expression during tumor initiation and progression [1]. They are responsible for the transformation that leads to cancer and further accelerate tumor progression [2]. The accurate identification of driver genes helps scientists understand the molecular mechanisms of cancer, enabling precise early diagnosis and targeted treatment. This leads to efficient detection and development of therapies that improve efficacy while minimizing harm to healthy cells. However, identifying cancer driver genes through traditional medical methods is not only time-consuming but also financially demanding. Large-scale cancer genomics projects like The Cancer Genome Atlas (TCGA) and the Catalogue of Somatic Mutations in Cancer (COSMIC) [3] provide the potential for using computational tools to detect cancer driver genes.

Initial methods such as MuSiC [4], MutSigCV [5], and ActiveDriver [6] based on frequency often assume that mutations in driver genes occur at a higher frequency compared to mutations in passenger genes. Consequently, they identify genes with statistically significant mutation rates. However, these methods are unable to identify drivers with low-frequency mutations owing to the challenge of establishing a reliable background model.

Advances in machine learning have led to powerful methods for identifying cancer driver genes. LOTUS [7] employs a multi-task learning framework that integrates mutation data with protein–protein interaction (PPI) networks to enable knowledge transfer across cancer types. DriverML [8] combines supervised machine learning with the Rao score test. It weights and quantifies how different mutation types affect gene function, and applies Monte Carlo simulation to determine the sample distribution of score statistics.

Graph neural network (GNN) is a deep learning model specifically designed for processing graph-structured data [9]. It can learn feature representations of nodes, edges, or entire graphs by aggregating the feature information of nodes and their neighbors, thereby improving the performance of node classification tasks. A biological network is a complex network structure that takes biological molecules as nodes and the interactions between these biological molecules as edges. It can intuitively show the relationship between the components of the biological system, and help to deeply understand the function and mechanism of the biological system as well as the occurrence and development of diseases.

Recent studies have developed GNN-based approaches for predicting cancer driver genes by integrating both multi-omic characteristics and biomolecular network information. EMOGI [10] is a GNN-based deep learning model. It integrates multi-omics pan-cancer data and PPI networks to predict cancer genes and can analyze their molecular mechanisms. MTGCN [11] is a multi-task learning model that optimizes node and link prediction tasks by leveraging a Bayesian task weighting scheme to automatically balance the two objectives. MCDHGN [12] constructs a multi-omics heterogeneous networks, manually identifies specific meta-paths within this network, and further aggregates information using these meta-paths.

However, the mentioned models only use undirected networks like PPI networks, which are unable to effectively capture the directed regulatory relationships between genes, and this is essential for accurate identification of driver genes. Zhang et al. proposed a method named DGMP [13] that combines a Directed Graph Convolutional Network (DGCN) with a Multilayer Perceptron (MLP) for cancer driver gene identification. DGMP learns both gene features and topological characteristics from gene regulatory networks and employs an MLP to adaptively weight gene features, thereby reducing the bias of DGCN toward topological information during learning. However, DGCN requires the use of multiple real-valued symmetric matrices (such as first-order and second-order adjacency matrices) to handle in- and out-neighbors separately, resulting in a complex model architecture. As a result, it is not effective at extracting node features from directed graphs with complex and noisy structures, such as gene regulatory networks. MF-GCN integrates three complementary network representations for the task of cancer driver gene prediction. It first employs the magnetic Laplacian to construct a directed topological graph. Then, it builds an attributed graph based on multi-omics features using a k-nearest neighbors algorithm. Finally, it captures consistent relationships between topological structure and node attributes through a consensus graph network. However, the directed gene networks utilized in MF-GCN are relatively limited, which restricts the amount of available gene network information. Moreover, its capability to capture critical biological information remains insufficient, ultimately impairing the model’s performance.

To address these limitations, we develop MDIGNN, which integrates directed gene networks from various databases with multi-omics data to construct a more informative directed graph network. We encode the directional information of the graph by introducing the magnetic Laplacian matrix. Its magnitude represents the presence of edges, while its phase indicates edge direction, thereby eliminating the complexity associated with DGCN’s requirement for multiple real-valued symmetric matrices to separately handle in- and out-neighbors. Additionally, our model incorporates a parameter to adaptively adjust the sensitivity to directional information, allowing it to capture structural patterns in directed graphs more flexibly. To further enhance the feature extraction capability, we introduce channel and spatial attention mechanisms to identify critical channels and locate semantically salient spatial regions. We evaluate MDIGNN’s performance by comparing it with state-of-the-art methods on a pan-cancer dataset. The results demonstrate that MDIGNN significantly outperforms these models. Moreover, by comparing the cancer driver genes predicted by MDIGNN with those in known databases and predictions from other models, we find that MDIGNN can effectively identify potentially novel driver genes that have not been previously reported, further demonstrating its efficacy in the identification of novel cancer driver genes.

In summary, the main contributions of our study are as follows:

(1) We integrate directed gene networks from multiple databases and combined them with multi-omics information to form a more comprehensive directed graph. Using the magnetic Laplacian matrix, we encode both the undirected connectivity of the graph and the directionality of edge. This allows full utilization of directional information in gene interactions.

(2) We introduce channel and spatial attention mechanisms to improve the model’s feature extraction capability. The channel attention mechanism highlights critical channels and effectively captures dependencies between channels, while the spatial attention module captures local information by focusing on key regions in the feature map.

(3) We propose a novel cancer driver gene prediction model based on directed gene networks and multi-omics data. It significantly outperforms existing methods in the field and demonstrates strong ability to identify potential cancer driver genes.

## 2. Results

### 2.1. Baselines

To assess the performance of MDIGNN, we conducted comparisons with the following state-of-the-art approaches (EMOGI [10], MTGCN [11], MODIG [14], HGDC [15], MRNGCN [16], MCDHGN [12], and DGMP [13]) in the field under ten 5-fold cross-validation.

EMOGI is an interpretable deep learning framework that utilizes Graph Convolutional Network (GCN) to predict cancer driver genes by integrating multi-omics pan-cancer data and PPI networks.

MTGCN employs a multi-task framework with a primary node prediction task and an auxiliary link prediction task. Additionally, it enhances gene features by integrating biological attributes with structural features from PPI network.

MODIG creates a multi-dimensional gene network and uses a multi-dimensional Graph Attention Network (GAT) encoder to learn within-dimension representations, while a joint learning module facilitates cross-dimension sharing and fusion.

HGDC builds an auxiliary network by applying graph diffusion to capture underlying structural similarities between nodes. Then, it employs a refined message propagation scheme to separate a gene’s ego-representation from the representations of its neighbors.

MRNGCN constructs three networks and creates a multi-view heterogeneous graph convolutional network with self-attention to learn from these networks.

MCDHGN constructs a heterogeneous network containing multi-omics data, manually selects meta-paths, and aggregates information based on them.

DGMP utilizes DGCN to learn multi-omics features of genes and topological characteristics of the gene regulatory network, then it employs MLP to mitigate DGCN’s inherent bias toward graph topological features.

### 2.2. Hyperparameters and Experimental Environment

Our computing infrastructure was a Linux system with 40 GB of NVIDIA A100 GPU. In our model, we employ the Adam optimizer with a learning rate of 1 × 10^−3^ and weight decay of 5 × 10^−3^ to refine the parameters. The model is trained using full-batch processing to preserve the complete graph structure, with a maximum of 3000 epochs and an early stopping mechanism that halts training if validation loss does not improve for 100 consecutive epochs. Table 1 provides the detailed search space for the hyperparameters of MDIGNN.

### 2.3. Performance on Pan-Cancer Driver Gene Prediction

We evaluated MDIGNN and baseline methods for pan-cancer driver gene prediction using a 5-fold cross-validation strategy. In each fold, the labeled data were partitioned into a training set (80%) and a test set (20%). All methods were assessed on the same gene expression and known cancer gene data.

**Figure 1:**
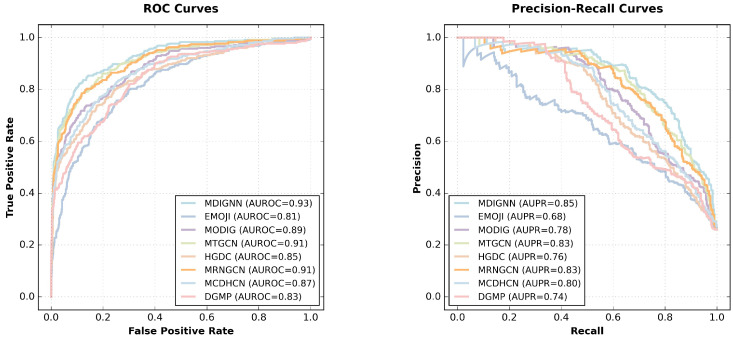
ROC curves and PR curves of different methods.

As shown in Table 2 and Figure 1, MDIGNN achieves the best performance in five out of seven key metrics. It achieves the highest scores in both AUROC (0.9302) and AUPR (0.8470), indicating a clear advantage in overall discriminative ability and effectiveness in handling imbalanced data. Additionally, it ranks first in accuracy (0.8720), MCC (0.6701), and precision (0.7959), reflecting robust predictions with a low false positive rate. It is also worth noting that the model’s specificity (0.9393) remains highly competitive, close to the best value in the table, which underscores its strong capability to correctly identify negative samples. However, while the model’s sensitivity (0.7420) remains relatively high, it is slightly lower than that of some other methods (EMOJI at 0.8028). This may stem from the model’s tendency to prioritize controlling false positives when balancing precision and recall, resulting in a somewhat conservative predictive bias under highly imbalanced data conditions.

Compared to the six baseline methods that use undirected graphs for the gene network, MDIGNN achieves better performance, indicating the importance of directional information in gene networks for pan-cancer driver genes prediction. When compared to DGMP, which also employs a directed gene network, MDIGNN utilizes a magnetic Laplacian-based graph neural network and incorporates both channel and spatial attention mechanisms, enabling more effective extraction of information from directed graphs and leading to improved performance.

### 2.4. Ablation Experiments

We performed an ablation study to investigate which components contribute to the exceptional performance of MDIGNN. The results are presented in Table 3.

It is evident that removing the feature enhancement module exerted the most notable influence on model performance, causing AUROC and AUPR to drop from 0.9302 and 0.8470 to 0.8789 and 0.7741, respectively. This indicates that incorporating structural properties of genes in the gene network aids in the accurate prediction of cancer driver genes.

To demonstrate the effectiveness of the magnetic Laplacian-based network used in this study in extracting directed graph information, we replaced it with a conventional GCN, resulting in reductions of 3.1% and 3.2% in AUROC and AUPR. This confirms that the magnetic Laplacian matrix enables MDIGNN to better capture directional information by encoding undirected geometric structure through magnitude and direction through phase.

We also evaluated the performance of the attention mechanism combinations by separately removing the channel attention module, the spatial attention module, and both together. The results show that the model demonstrates its peak predictive performance when both attention mechanisms are employed, indicating that MDIGNN effectively prioritizes critical channels and locates semantically important spatial regions through attention, improving feature extraction capability. Furthermore, integrating additional directed gene networks and incorporating system-level features of genes both contributed to improved model performance.

Overall, each module included in MDIGNN collectively provide outstanding prediction performance, and removing any module will compromise the model’s predictive ability.

### 2.5. Performance on Identifying Novel Cancer Genes

**Figure 2:**
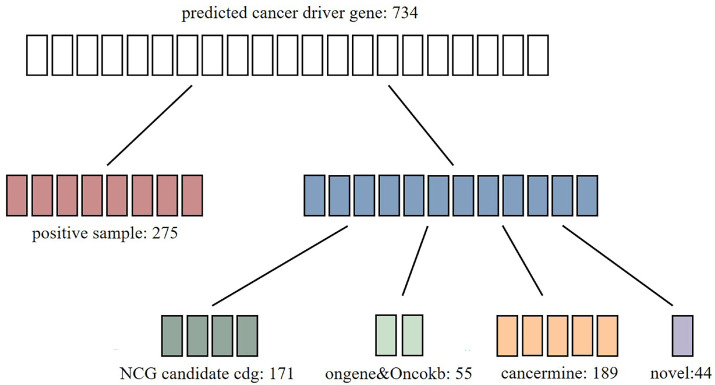
Breakdown of known and novel cancer driver genes predicted by MDIGNN.

After training on the entire dataset, prediction scores have been obtained for every gene node. Employing a threshold of 0.9, we identified 734 genes as potential cancer drivers. Of these, 275 genes overlap with the known positive set. To investigate the novel predictions, we analyzed the remaining 459 genes and found that 55 are supported by existing evidence in the OncoKB [17] and OncoGene database, suggesting some evidence of their association with cancer. Next, we employed Cancer Miner [18], a text-mined database integrating evidence on driver genes, oncogenes, and tumor suppressor genes. This tool identified 189 genes as high-confidence candidates. Figure 2 shows that 94% of the candidates predicted by MDIGNN are supported by existing publications or databases. The remaining 44 genes (6%) are novel candidates without direct research evidence requiring future validation.

**Figure 3:**
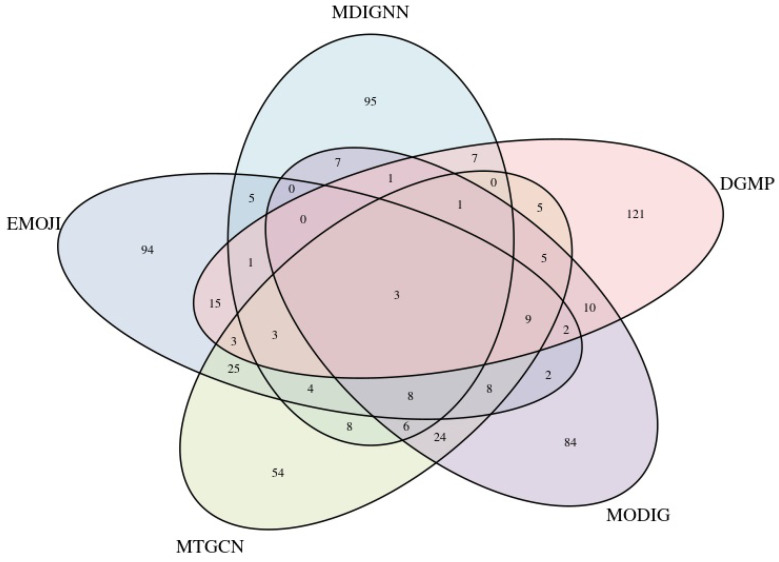
Venn diagram of the overlap between the MDIGNN and several other methods.

As shown in Figure 3, after excluding known driver genes, comparative analysis against several established methods (EMOGI, MODIG, MTGCN, and DGMP) reveals that over half of MDIGNN’s novel driver gene predictions are unique. This suggests that MDIGNN can predict novel candidates that other methods can not identify.

### 2.6. Analysis of Novel Cancer Genes

**Figure 4:**
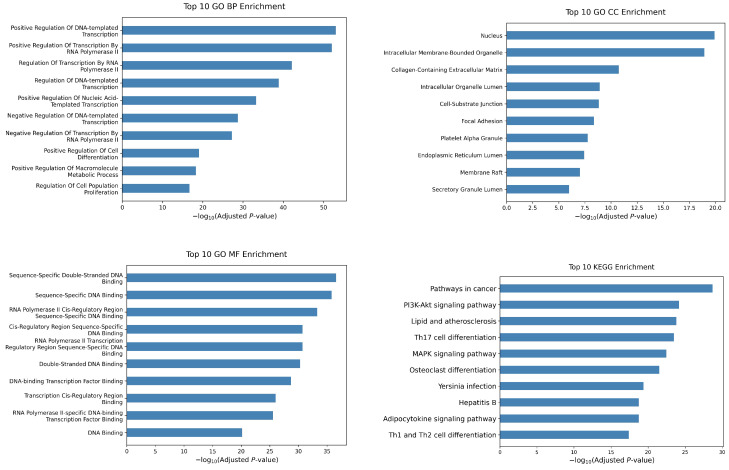
Enrichment analysis of the top 300 genes predicted by our model after deleting those with known labels.

To further assess our model’s capacity to identify novel cancer driver genes, we conducted enrichment analysis on the top 300 genes after removing those with known labels. The results are illustrated in Figure 4. For biological process, the candidate genes are significantly enriched in “Positive Regulation of DNA-templated Transcription”, “Positive Regulation of Transcription By RNA Polymerase II”, and pathways involved in the regulation of cell differentiation and proliferation. For cellular components, the most significantly enriched GO terms are “Nucleus”, “Intracellular Membrane-Bounded Organelle”, and “Collagen-Containing Extracellular Matrix”. For molecular functions, key enriched terms include “Sequence-specific double-stranged DNA Binding”, “RNA Polymerase II cis-regulatory region sequence-specific DNA binding”, and “Sequence-Specific DNA Binding”, highlighting critical dysregulation in transcriptional control. As for KEGG enrichment analysis, the most significantly enriched pathway is “Pathways in cancer”, which refers to the critical cell signaling pathways that play a role in cancer’s onset, progression, and metastasis. The second most enriched pathway is the PI3K-Akt signaling pathway, which has a well-established link to cancer and and contributes significantly to its development and progression [19]. The potential driver genes we predict are also significantly enriched in “Th17 Cell Differentiation”, “MAPK Signaling Pathway”, and “Th1 and Th2 Cell Differentiation”. In cancer, Th17 cells can promote tumor progression through immune suppression and angiogenesis. However, they can also recruit immune cells or transform into a Th1 phenotype to secrete IFN-γ, thereby mediating anti-tumor immune responses [20]. The Ras/Raf/MEK/ERK pathway is a key cascade among MAPK pathways for its critical function in tumor cell survival and development [21]. Th1 cells promote cytotoxic responses by producing IFN-γ [22], while Th2 cells and regulatory T cells exert immunosuppressive effects that support tumor growth [23]. Several other pathways also have certain degrees of association with cancer [24,25,26]. In summary, the top-ranked genes identified by MDIGNN show significant enrichment across pathways related to cancer.

In addition, analysis of the top ten genes after filtering out known drivers is shown in Table 4. These genes playing roles in tumor processes such as proliferation and metastasis, as well as in cellular physiology such as apoptosis and metabolism. They are also involved in clinical diagnostics, prognosis, and therapeutic targeting. These genes may affect cancer prognosis or participate in tumor-related cellular activities, making them key molecules in cancer research. This further demonstrates our model’s capability to discover novel cancer driver genes.

## 3. Discussion

In this study, we propose a deep learning model that integrates directed gene networks and multi-omics data to identify cancer driver genes. Compared to other models, MDIGNN effectively extracts gene network information from directed graphs using a graph neural network based on the magnetic Laplacian matrix combined with the attention mechanism. MDIGNN significantly outperforms state-of-the-art methods on a pan-cancer driver gene dataset. Additionally, we compared the potential driver genes output by our model with known databases and gene lists from other models. Through the enrichment analysis and literature research, we demonstrate our model’s capability to identify novel cancer driver genes.

In our future work, we plan to further enrich node and edge features in the graph. While the current model only integrates multi-omics data and gene interaction information, we intend to incorporate more biologically meaningful features for specific prediction tasks. The reliability of the selected networks and the effectiveness of data augmentation strategies significantly affect the performance of our model, so we will explore more efficient data augmentation techniques in the future. Furthermore, our current model underperforms on datasets with limited known positive samples. To address this, we will focus on improving its capability in predicting cancer driver genes for specific cancer types with limited positive samples.

## 4. Materials and Methods

### 4.1. Datasets

We use multi-omics data obtained from TCGA. Similar to EMOGI, we focus only on cancer types for which multi-omics data available in both tumor and normal tissues. For each cancer type, we calculated gene mutation rate, gene differential DNA methylation rate, and gene differential expression rate. The gene mutation rate for a given cancer type is calculated as the average frequency of single nucleotide variations and copy number aberrations across all analyzed samples. The differential DNA methylation rate for each gene is defined as the average difference in methylation signals between tumor samples and their matched normal counterparts, computed across all samples within that cancer type. The formula is as follows:

(1) $$dm_{i}^{c}=\frac{1}{|S_{c}|}\sum_{s\in S_{c}}\left(\beta_{i}^{t}-\beta_{i}^{n}\right)$$

where $dm_{i}^{c}$ is the DNA methylation expression of gene *i* in cancer type *c*. $S_{c}$ is the sample set of cancer *c*. $\beta_{i}^{t}$ and $\beta_{i}^{n}$ are the methylation signals in cancer and matched normal samples. The differential expression level of a gene is calculated as the log2 fold change between its expression in a tumor sample and the corresponding normal sample. This value is then averaged across all samples within a given cancer type. In this way, we construct a 48-dimensional biological feature vector for each gene.

We adopt the system-level properties (SYS) from HGDC [15], which represent six global gene properties not explicitly related to cancer. Specifically, systems-level properties include ten specific properties across six categories, such as gene duplication, gene essentiality, tissues expressing gene, topological properties of biomolecular network, protein complexes, and miRNA interactions. Gene duplication is represented by a binary feature indicating whether the gene is an ohnolog, based on the assumption that canonical driver genes are less likely to have duplicated loci. Gene essentiality is captured by two features: the continuous percentage of cell lines in which the gene is essential and a binary indicator of essentiality, corresponding to the assumption that driver genes are more likely to be essential. Gene expression is characterized by the continuous number of healthy tissues expressing the gene, reflecting the assumption of broader expression. Topological properties of biomolecular networks encompass four features: continuous network degree, a binary hub indicator, continuous network betweenness, and continuous clustering coefficient, under the assumption that driver genes have distinct connectivity patterns. Protein complexes are represented by the continuous number of complexes the protein participates in, based on the assumption of greater complex involvement. Finally, miRNA interactions are indicated by the continuous number of targeting miRNAs, corresponding to the assumption of increased miRNA regulation. These six properties, integrated from sysSVM2 [38], collectively form a 10-dimensional vector used to characterize and distinguish cancer driver genes.These are then integrated with multi-omics data to construct a 58-dimensional feature vector.

We use gene networks from various databases including KEGG [39], RegNetwork [40], TRRUST [41], MOTIFMAP [42], HTRIdb [43], EVEX [44], JASPAR [45], CHEA [46], ENCODE [47], TRANSFAC [48], and PPI networks including CPDB [49], STRING [50], IRefIndex [51], and PCNet [52]. We preprocess the data using the multiple specialized methods in DGP-AMIO [53]. To ensure high-quality interactions, we apply confidence score thresholds to each database: >0.5 for CPDB, >0.85 for STRING, and only retained high or medium confidence regulations for RegNetwork. For IRefIndex, which lacks confidence scores, we only keep binary interactions. As for KEGG, we use KEGGREST to download 345 human pathways. These pathways are then transformed into directed graphs. Subsequently, we employ the KEGGgraph package in R to merge these graphs, generating a single directed graph including all the downloaded pathways.

We use the same positive samples as MTGCN, which originate from The Network of Cancer Genes (NCG) [54] as well as high-confidence genes identified from the literature sources. We recursively remove genes from all genes included in the NCG, COSMIC, the Online Mendelian Inheritance in Man (OMIM) database, and KEGG cancer pathways to obtain a negative sample list. After the mentioned preprocessing, the pan-cancer dataset used in this study includes 796 positive samples and 2187 negative samples.

Following the preprocessing steps described above, we constructed a large-scale directed gene regulatory network comprising 13,212 nodes and 1,610,899 directed edges. Based on the existing knowledge, the nodes were annotated as follows: 796 are known cancer driver genes, 2187 are known non-cancer driver genes, and the cancer-associated status of the remaining 10,229 genes is currently unknown.

### 4.2. Framework of MDIGNN

**Figure 5:**
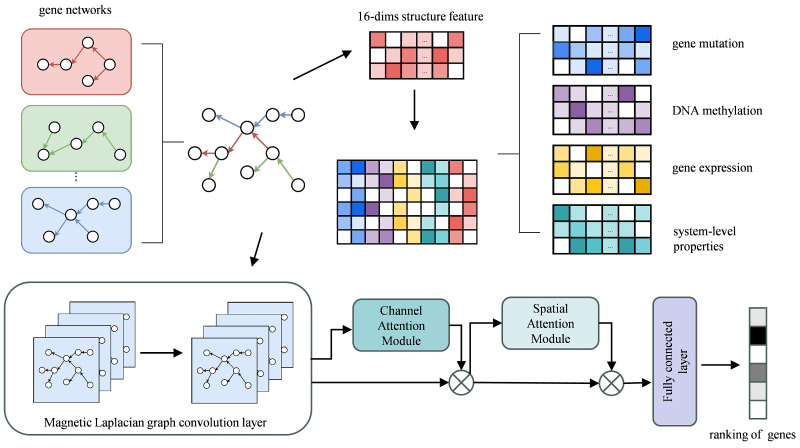
The framework of MDIGNN. MDIGNN is composed of four primary modules. First, this study combines gene networks from different databases to form a directed network. Then, MDIGNN uses a magnetic Laplacian-based graph neural network to encode directional information. Next, MDIGNN separately incorporates channel attention modules and spatial attention modules to enhance the feature extraction capability. Finally, MDIGNN uses fully connected layers to generate the probability scores for cancer driver gene classification.

MDIGNN is a GNN-based model for cancer driver gene prediction that integrates directed gene networks and multi-omics data. Figure 5 illustrates the overall framework of MDIGNN. First, we integrate databases from multiple sources to form a more comprehensive gene network. Additionally, we incorporate topological features to enrich the characteristics of gene nodes. Next, we introduce the magnetic Laplacian matrix, where the matrix’s magnitude encodes the undirected connectivity structure and the phase encodes edge directional information. After this, we add channel attention and spatial attention modules. Channel attention enhances semantic information by adaptively adjusting the importance of feature channels, while spatial attention focuses on key areas to optimize spatial response. Finally, we use a fully connected layer to output the probability of a gene being a cancer driver gene.

### 4.3. Feature Enhancement

We use the same feature enhancement method as MTGCN. Specifically, we employ the Node2Vec model to extract global structural features from the graph. Node2Vec is an unsupervised technique for deriving node embeddings by simulating random walks across the graph, which are then processed using the Skip-Gram model to generate embeddings. We set the embedding dimension to 16, the walk length to 80, and the context size to 5, performing 10 walks per node. This process enables us to capture 16-dimensional topological features of the genes. We then concatenate these topological features with the biometric features, resulting in a 74-dimensional initial feature matrix for each gene.

### 4.4. Constructing Graph Magnetic Laplacian

Gene regulatory networks are directed, but most existing methods treat them as undirected graphs. These methods ignore the direction information, which is essential for representing the architecture of gene regulatory networks and signaling pathways. To overcome this limitation, inspired by MagNet [55], we extract the geometric and directional information of directed graphs by constructing the complex Hermitian adjacency matrix, and then deriving the magnetic Laplacian operator.

We model the gene regulatory network as a directed graph G=(V,E), where *V* is the set of *N* gene nodes and E⊆V×V is the set of directed edges that capture interactions between them. An edge from *u* to *v* is represented by the pair (u,v)∈E. The structure of the directed gene regulatory network is represented by an asymmetric adjacency matrix *A*, where the entry A(u,v) equals 1 for a directed edge from *u* to *v* ((u,v)∈E), and 0 otherwise.

To mitigate the asymmetry caused by directional differences in the original directed graph adjacency matrix, we calculate the average of the connections between two directions. This results in a symmetric matrix, as defined in Equation (2), which provides the fundamental connection information. We also introduce the phase matrix $\Theta^{\left(q\right)}\left(u,v\right)$ to capture the directional information in a directed graph, which is defined as (3).

(2) $$\begin{array}{cc} S(u,v) & =\frac{1}{2}\left(A\left(u,v\right)+A\left(v,u\right)\right),\;1\leq u,v\leq N \end{array}$$

(3) $$\Theta^{\left(q\right)}\left(u,v\right)=2\pi q\left(A\left(u,v\right)-A\left(v,u\right)\right),\;q\geq 0$$

The parameter *q* is used to adjust the degree or sensitivity of encoding directional information. Larger values of *q* will make the phase matrix more sensitive to differences in edge directions, meaning that the values in the phase matrix will vary more significantly with changes in direction. 2π ensures that the phase $\Theta^{\left(q\right)}$ fully spans the range [0,2π) as *q* varies, thereby allowing the model to effectively utilize the entire complex unit circle to represent directional asymmetry [55].

The complex Hermitian adjacency matrix $H^{\left(q\right)}$ is constructed from the symmetrized adjacency matrix *S* and the phase matrix $\Theta^{\left(q\right)}$ as follows:

(4) $$H^{\left(q\right)}=S⊙\exp\left(i\Theta^{\left(q\right)}\right)$$

where ⊙ denotes element-wise multiplication. *i* denotes the imaginary unit, converting the directional differences into phase shifts on the complex unit circle. The element values and phases of the $H^{\left(q\right)}$ matrix respectively contain information about the existence and direction of edges in the graph. Since $\Theta^{\left(q\right)}$ is an skew-symmetric matrix, $H^{\left(q\right)}$ is a Hermitian matrix.

Then, we define normalized magnetic Laplacian as:

(5) $$L^{\left(q\right)}=I-D^{-1/2}SD^{-1/2}⊙\exp\left(i\Theta^{\left(q\right)}\right)$$

where *I* is the identity matrix, *D* is the degree matrix corresponding to the symmetrized adjacency matrix *S*, representing the sum of connection numbers of each node, which is used to balance the connection strength between nodes. This matrix is Hermitian and positive-semidefinite, ensuring real non-negative eigenvalues and an orthonormal eigenbasis. Critically, $L^{\left(q\right)}$ preserves directional asymmetry: unidirectional edges induce phase differences in eigenvectors, while bidirectional edges retain zero phase shifts. The eigenvalues of $L^{\left(q\right)}$ are bounded within [0,2], similar to the traditional normalized Laplacian. Adjusting *q* allows adaptive control over directional sensitivity. For example, q=0 recovers the undirected Laplacian, whereas q>0 emphasizes edge directionality.

We defined the specific calculation method of the convolution operation in the network as

(6) $$Yx=\sum_{k=0}^{K}\theta_{k}T_{k}\left({\tilde{L}}^{\left(q\right)}\right)x$$

where $T_{k}$ is the Chebyshev polynomial, and $\theta_{k}$ are learnable parameters. The weights for these polynomials of different orders are optimized automatically during training. Spectral convolution is implemented by approximating the filter with Chebyshev polynomials. This avoids the need for explicit eigen decomposition and thus reduces the computational complexity.

We define the feature matrix of the *l*-th layer as

(7) $$X^{\left(\ell \right)}=\sigma \left(X^{\left(\ell -1\right)}W_{\mathrm{self}}^{\left(\ell \right)}+{\tilde{L}}^{\left(q\right)}X^{\left(\ell -1\right)}W_{\mathrm{neigh}}^{\left(\ell \right)}+B^{\left(\ell \right)}\right)$$

where $X^{\left(\ell -1\right)}$ is the feature matrix of the (ℓ−1)-th layer, which serves as the input for the calculation of the current layer. $W_{\mathrm{self}}^{\left(\ell \right)}$ and $W_{\mathrm{neigh}}^{\left(\ell \right)}$ are learnable weight matrices. $W_{\mathrm{self}}^{\left(\ell \right)}$ adjusts the weights of the node’s own features, enabling the network to focus on the unique attribute information of each node. $W_{\mathrm{neigh}}^{\left(\ell \right)}$ is used to regulate the influence of the neighbor nodes’ features on the current node’s features, helping the network capture the local structural information around the node. $B^{\left(\ell \right)}$ is the bias matrix, which introduces additional learnable parameters to the entire convolution calculation result. Furthermore, as our filters are complex, we employ a complex version of ReLU.

### 4.5. Attention Module

Then, we introduce an attention mechanism into the model. We combine channel attention and spatial attention, applying them sequentially to the feature matrix to adaptively optimize the feature representation. Given input feature matrix *X*, the optimized feature matrix $X^{\prime \prime }$ can be described as follows:

(8) $$X^{\prime }=X⊙M_{c}\left(X\right)$$

(9) $$X^{\prime \prime }=X^{\prime }⊙M_{s}\left(X^{\prime }\right)$$

where $M_{c}$ is the channel attention map and $M_{s}$ is the spatial attention map.

#### 4.5.1. Channel Attention Module

The channel attention mechanism emphasizes important channels, capturing inter-channel dependencies. The channel attention map $M_{c}$ is computed using (10).

(10) $$M_{c}\left(X\right)=\sigma \left(\mathrm{MLP}\left(\mathrm{AvgPool}(X)\right)\right)$$

where σ is the sigmoid function. The input feature are subjected to average-pooling operation which aggregates global spatial information by calculating the mean across spatial dimensions. The pooling results are sent into a shared multilayer perceptron (MLP) for feature transformation, which reduces parameter overhead while exploring the dependencies between channels.

#### 4.5.2. Spatial Attention Module

The spatial attention module captures local information, focusing on important regions within the feature map. The spatial attention map $M_{s}$ is defined as follows:

(11) $$M_{s}\left(X\right)=\sigma \left(f^{7}\left(\left[\mathrm{AvgPool}(X);\mathrm{MaxPool}(X)\right]\right)\right)$$

where σ is the sigmoid function, and $f^{7}$ represents a convolution operation with a filter size of 7. We employ average pooling and max pooling to extract spatial features along the channel dimension. The two sets of pooled features are then concatenated, and spatial dependencies are learned through a convolutional layer to generate raw attention scores. Finally, we apply a sigmoid activation to obtain the spatial attention map.

## Figures and Tables

**Table 1:** Architecture search space for MDIGNN.

Hyperparameter	Supernet Size
num of filters	64
K for cheb series	1
layer	2
dropout rate	0.5
learning rate	1 × 10^−3^
weight decay	5 × 10^−3^

**Table 2:** Performance comparison on pan-cancer driver gene prediction.

Method	AUROC	AUPR	Accuracy	Sensitivity	Specificity	Precision	MCC
EMOJI	0.8123	0.6758	0.7139	0.8028	0.6825	0.4711	0.4287
MODIG	0.8941	0.7818	0.8533	0.6423	0.9276	0.7575	0.6029
MTGCN	0.9102	0.8334	0.8650	0.7296	0.9127	0.7464	0.6471
HGDC	0.8468	0.7620	0.7545	0.6681	0.8101	0.6978	0.5654
MRNGCN	0.9061	0.8265	0.8672	0.7155	0.9206	0.7605	0.6491
MCDHCN	0.8703	0.7971	0.8437	0.5915	0.9325	0.7554	0.5708
DGMP	0.8275	0.7413	0.8239	0.4873	0.9425	0.7489	0.5028
MDIGNN	0.9302	0.8470	0.8720	0.7420	0.9393	0.7959	0.6701

The reported performance metrics are the mean values obtained from ten repetitions of 5-fold cross-validation.

**Table 3:** The ablation results of MDIGNN.

Method	AUROC	AUPR
Use GCN layer	0.9012	0.8136
Only PPI	0.9202	0.8410
Only KEGG	0.9244	0.8394
Only RegNetwork	0.9255	0.8408
Without sys	0.9229	0.8334
Without feature enhancement	0.8789	0.7741
Without edgedropout	0.9201	0.8396
Without channel attention	0.9276	0.8402
Without spatial attention	0.9262	0.8420
Without attention	0.9279	0.8397
MDIGNN	0.9302	0.8470

**Table 4:** Top 10 ranked potential cancer driver genes predicted by MDIGNN and supporting evidence from the literature.

Gene	Function
*FOXL1*	The expression of *FOXL1* is typically downregulated in kidney and breast cancer, and its high expression is often associated with better prognosis [27,28].
*TFAP2A*	The mRNA expression level of *TFAP2A* is elevated, and genetic alterations of this gene are commonly found in many types of cancer [29].
*HOXA5*	Overexpression of *HOXA5* is linked to apoptosis in various cancers, including breast, lung, and cervical cancer [30].
*NFIC*	*NFIC* exhibits a complex dual role in cancers, functioning as either a tumor suppressor or an oncogenic factor [31,32].
*FOXA2*	*FOXA2* plays various biological roles in the development and progression of tumors by regulating the expression of multiple genes involved in core pathways of tumorigenesis and chemotherapy resistance [33].
*EPHB2*	*EPHB2* dysregulation has been noted in a range of cancer types, where it shows important diagnostic and prognostic significance [34].
*TFAP2C*	The role of *TFAP2C* in tumor proliferation, invasion, modulation of the immune microenvironment, and treatment response makes it a candidate for both prognosis and therapy [35].
*PPARD*	*PPARD* aids cancer cell survival under chemotherapy conditions by regulating cell metabolism and stress responses [36].
*USF2*	*USF2* is supposed to play significant roles in metabolism, tissue protection, and tumor development.
*NANOG*	*NANOG* is abnormally overexpressed in diverse cancer and is associated with tumor progression, metastasis, and prognosis [37].

## Data Availability

The original data presented in the study are openly available on 12 November 2025 at https://github.com/xvincyx/MDIGNN.
